# Cellular mechanisms underlying pituitary adenylate cyclase activating polypeptide-stimulated secretion in the adrenal medulla

**DOI:** 10.1042/BST20231326

**Published:** 2024-12-10

**Authors:** Nicole A. Bell, Xiaohuan Chen, David R. Giovannucci, Arun Anantharam

**Affiliations:** Department of Neurosciences, University of Toledo, Toledo, OH 43614, U.S.A.

**Keywords:** adrenal medulla, calcium signaling, exocytosis, PACAP

## Abstract

The adrenal medulla is a key effector of the sympathetic nervous system in the periphery. Its primary function is to translate variations in sympathetic activity into hormone outputs that modify end organ function throughout the body. These hormones include epinephrine, norepinephrine, and a variety of vasoactive peptides. Hormone secretion occurs when neurotransmitters, delivered by sympathetic nerves, bind to, and activate receptors on adrenomedullary chromaffin cells. In this context, two neurotransmitters of particular importance are acetylcholine (ACh) and pituitary adenylate cyclase activating polypeptide (PACAP). PACAP, discovered initially as a secretagogue in the hypothalamus, is now appreciated to provoke a strong secretory response from chromaffin cells *in vitro* and *in situ*. However, the cellular mechanisms underlying PACAP-stimulated secretion are still poorly understood. In the sections below, we will summarize what is known about the actions of PACAP in the adrenal medulla, discuss recent advances that pertain to the PACAP signaling pathway, and highlight areas for future investigation.

## The adrenal medulla: innervation, organization, and function

The adrenal glands are situated above the kidneys in the perirenal space [1,2]. ‘Medulla’ refers to the central region of the adrenal gland, which is surrounded by tissue of distinct mesodermal origin, the adrenal cortex [2]. In contrast with cortical cells, chromaffin cells, which constitute the principal secretory units of the medulla, share, at least in part, a common lineage with sympathetic neurons [3,4]. The secretion of hormones from the adrenal medulla is controlled by descending nerve fibers which pass into the gland, referred to as splanchnic nerves [5,6]. At synaptic sites in the medulla, neurotransmitters, including acetylcholine (ACh) and pituitary adenylate cyclase activating polypeptide (PACAP), are released directly onto adrenomedullary chromaffin cells [7–10]. The subsequent activation of receptors on chromaffin cells causes catecholamines and peptide hormones to be delivered into the circulation via the process of dense core vesicle exocytosis. The physiological effects of these agents are widespread. They cause, for example, the vasoconstriction of blood vessels in the periphery and dilate airways in the lung; they speed the heart rate and increase the force of ventricular contractions [11,12]. In addition, epinephrine-induced activation of β-adrenergic receptors (βARs) in the liver causes gluconeogenesis to supply glucose as an energy source [13,14]. Together, these events enhance cardiovascular tone, increase delivery of oxygen to organs, and augment glucose availability to muscles to support the ‘fight-or-flight’ response [15].

A knowledge of the rates of splanchnic discharge at rest and during stress is essential to appreciating the function of the adrenal medulla and the mutability of its secretory output [2]. In humans, splanchnic discharge rates have been estimated to dwell at ∼1 Hz at rest and between 10 and 20 Hz when the sympathetic nervous system is fully activated [16,17]. Animal models have also been useful in assessing how sympathetic activity might vary in response to physiological stressors. For example, in rabbits exposed to intravenous insulin — a stimulus known to strongly stimulate epinephrine secretion from the adrenal medulla — firing frequency of sympathetic nerves rises from a basal rate of ∼10 Hz to a peak of 40 Hz [18]. In rats, basal firing rates are estimated to be in the 2–5 Hz range [19]. However, direct injection of glutamate into the rostral ventrolateral medulla — a brain region gating sympathetic outflow to the adrenal medulla in the periphery — causes a several-fold increase in splanchnic nerve activity [19–21]. Among the many compelling outcomes of these studies is the observation that discharge rates may depend on whether splanchnic nerves are targeting epinephrine-secreting or norepinephrine-secreting chromaffin cells [19]. Chromaffin cells are thought to produce and secrete either epinephrine or norepinephrine, but not both [22,23].

In the adrenal medulla, as in other secretory tissue, exocytosis has a strong Ca^2+^ dependence [24]. Thus, in order for catecholamines to be released from chromaffin cells, receptor activation must lead to elevations in cytosolic Ca^2+^. Exactly how this occurs varies depending on the identity of the receptor that is activated and the signaling pathway to which it is coupled. For example, it has long been known that activation of nicotinic ACh receptors (nAChRs) causes a membrane depolarization which opens voltage-gated Ca^2+^ channels [25,26]. Stimulation of nicotinic, muscarinic, and PAC1 receptors has also been reported to cause Ca^2+^ release from internal stores [27,28]. This form of Ca^2+^ release, which itself is Ca^2+^-dependent, can occur through ryanodine receptors (RyRs) or inositol trisphosphate receptors (IP3Rs) on the ER [28]. Regardless of the exact mechanism by which intracellular Ca^2+^ is raised, for exocytosis to occur the signal must then be transduced by Ca^2+^-sensitive proteins that drive the fusion of dense core vesicles with the plasma membrane (PM) [29].

## PACAP functions as a neurotransmitter in the central and peripheral nervous systems

The PACAP gene encodes a 176 amino acid prepropeptide which is processed into two forms, PACAP-38 and PACAP-27, each with approximately equal affinity for the major PACAP-specific receptor, PAC1R [30]. Additional receptors for PACAP with a broad tissue distribution profile have since been identified (VPAC1, VPAC2), however PACAP has a substantially higher affinity for PAC1 than either VPAC1 or VPAC2 receptors [31]. The actions of PACAP in human and rat chromaffin cells are inhibited by PACAP (6–38) — a PAC1R-specific antagonist — suggesting PAC1R is the relevant receptor for PACAP-evoked secretion in the adrenal medulla [32–34].

PACAP was originally identified as a peptide in the hypothalamus and shown to stimulate adenylyl cyclase activity in the anterior pituitary gland [35]. It was subsequently shown to be a powerful regulator of the hypothalamic-pituitary-adrenal axis, with its expression being necessary for the release of corticotropic-releasing hormone, adrenocorticotropic hormone, and corticosterone [36]. More recently, evidence has emerged for a connection between enhanced PACAP secretion, single nucleotide polymorphisms in the PAC1 receptor, and post-traumatic stress disorder [37–39]. PACAP-ergic signaling has been implicated in age-related diseases, migraines, and even sudden infant death [40–42]. However, the remainder of our attention, below, will be directed to delineating the effects of PACAP on adrenal chromaffin cells.

Shortly after the isolation of the PACAP neuropeptide in the CNS, it was applied to rat adrenal chromaffin cell cultures and shown to cause an increase cAMP production and catecholamine secretion [43]. PACAP was subsequently demonstrated to elicit secretion from frog, cow, dog, pig, and mouse chromaffin cells [44–48]. Definitive evidence that PACAP has a regulated role in stress-evoked secretion from the adrenal medulla was first provided by Eiden and colleagues, who showed that PACAP-deficient mice could not properly counter-regulate plasma glucose levels in response to insulin-induced hypoglycemia [48]. The cause of this dysregulation was revealed to be inadequate epinephrine secretion from the adrenal medulla [48]. Subsequent experiments by Eiden and Smith suggested a mechanism by which to link the defective *in vivo* epinephrine secretion phenotype with PACAP expression at the synapse. In fact, it appeared that PACAP was preferentially released from splanchnic nerves during burst activity, *in situ*, thereby causing persistent release of catecholamines from chromaffin cells in the adrenal medulla [33,49,50]. Out of these studies emerged the idea that PACAP may function as an ‘emergency response’ neurotransmitter, perhaps even supplanting cholinergic transmission under conditions associated with strong sympathetic activity [51,52].

## The role of voltage-gated Ca^2+^ channels in the PACAP pathway

It was apparent from the earliest studies on PACAP in the adrenal medulla that the exocytosis it causes requires Ca^2+^ influx [9]. Yet, unlike ACh, PACAP does not cause adrenal chromaffin cells to fire action potentials, and in fact, only causes a small depolarization of the membrane potential [26,33,53]. Given the requirement of external Ca^2+^ for secretion, how, then, does Ca^2+^ enter the cell?

Chromaffin cells have the potential to express all of the major forms of voltage-gated Ca^2+^ channels: the L-, N-, P/Q-, R-, and T-type channels have all been identified in this system [54,55]. Exactly which channels are expressed appears to depend on the developmental stage and the species in question. Based on its relatively small effects on the membrane potential, it seems most likely that PACAP stimulation provokes Ca^2+^ entry through a low-voltage-activated channel — one that might regulate excitability at or near the resting membrane potential (∼−50 mV [26,56–59]). With this requirement in mind, it has been suggested that PACAP activates a T-type channel for Ca^2+^ entry [33,60]. T-type channels open readily at negative potentials and conduct a transient Ca^2+^ current [61]. However, T-type channels are typically expressed at low levels in the rodent chromaffin cells where much of the recent characterization of PACAP effects have been performed [54]. Instead, multiple lines of evidence support the involvement of L-type channels, of either the Ca_v_1.2 or Ca_v_1.3 variety, in regulating the exocytotic response to PACAP stimulation. It is known, for example, that these channel isoforms provide almost 50% of the total Ca^2+^ current in rat and mouse chromaffin cells [59,62]. Moreover, PACAP-stimulated Ca^2+^ influx, and exocytosis, are sensitive to L-type channel inhibition. Nifedipine — a potent and specific blocker of L-type channels [55,63] — reduces inward currents and cytosolic Ca^2+^ signals evoked by PACAP in a dose-dependent manner [53,64]. In addition, concentrations of nifedipine that reduce PACAP-evoked cytosolic Ca^2+^ elevations, also disrupt exocytosis [64].

An interesting feature of chromaffin cells, and one with potentially important implications for secretion, is that they exhibit spontaneous, repetitive spiking. This ‘pacemaking’ activity occurs at frequencies ranging from 0.1 to 2 Hz [26,53,56,62,65]. This is true whether activity is measured in cells in culture or in slices [58,59,62,65–67]. Such observations suggest chromaffin cells are more than simple ‘relay elements’ whose activation depends solely on presynaptic stimulation [56]. But what causes the pacemaking? Although the mechanisms underlying the spontaneous action potentials in chromaffin cells are not fully resolved, several reasonable models have been proposed based on the properties of channels known to be active at or near the resting membrane potential of the cell. Many of these models have converged on L-type Ca_v_1.3 channels and large conductance, Ca^2+^-activated K^+^ (BK) channels as being especially important [62,66,68]. Ca_v_1.3 may function as a key regulator of spontaneous firing for reasons having to do with its intrinsic biophysical properties. These reasons include: (1) it activates at a more negative voltage than Ca_v_1.2; and, (2) it inactivates slowly during prespike, subthreshold depolarization [66]. Remarkably, in the absence of Ca_v_1.3, only 30% of chromaffin cells exhibit spontaneous spiking, compared with 80% of wild-type chromaffin cells [66]. BK channels, on the other hand, are likely not critical for generating spontaneous action potentials, but important in modulating action potential shape, stabilizing the repolarization phase, and setting firing frequency [56,65,66]. Thus, Ca_v_1.3 and BK channels are thought to function together to set major parameters of spiking activity, including their incidence, form, and frequency.

PACAP can change the frequency as well as the pattern (i.e. where tonic spiking becomes ‘bursts’ [56]) of chromaffin cell pacemaking activity [53]. Chromaffin cells exposed, even briefly (20 s), to PACAP increase their firing frequency from ∼1 to 1.5 Hz [53]. Two questions arise from this observation: (1) what are the mechanisms underlying the effects of PACAP on spontaneous action potentials?; and, (2) what are the implications of this modulation for exocytosis? Again, no mechanism is yet firmly attributable to this phenomenon. However, it was previously demonstrated that phosphodiesterase inhibition — which elevates cAMP levels and, thereby, cAMP-dependent signaling — up-regulates Ca_v_1.3 activity and effectively doubles the rate of spontaneous spiking in chromaffin cells [59]. As we will discuss later, the PACAP signaling pathway relies on cAMP as a critical intermediate; cAMP may then activate downstream signaling molecules, such as PKA and/or protein kinase C (PKC) that increase spiking frequency by acting directly, or indirectly, on Ca_v_1.3.

An important issue when considering PACAP-dependent modulation of spontaneous action potentials is its impact on exocytosis. In dissociated mouse chromaffin cells, a single action potential has been estimated to release between one and two vesicles [69]. The number of fusion events triggered by an action potential in adrenal slices might be higher, perhaps even as high as seven vesicles [69]. One can therefore easily conceive of the increased levels of intracellular Ca^2+^ that accompany the transition from tonic to action potential bursts as having a significant impact on the probability of vesicle fusion and catecholamine release.

## PACAP causes Ca^2+^ release from internal stores

Although their details may not always align, chromaffin cell studies are concordant on two key issues: (1) PACAP causes a long-lasting secretory response; and, (2) the PACAP secretory response is triggered by a persistent increase in intracellular Ca^2+^. By most accounts, the Ca^2+^ signal evoked by PACAP requires Ca^2+^ influx from the extracellular space [9,33,47,53,64]. However, the secretory response also appears to be sustained by Ca^2+^ mobilization from internal stores. Indeed, the involvement of internal Ca^2+^ stores has been suggested effectively from the time PACAP was first discovered to cause catecholamine secretion from the adrenal medulla. Experiments in PC12 cells, bovine chromaffin cells, rat chromaffin cells, and even human fetal chromaffin cells have either implied, or shown, PACAP to harness internal Ca^2+^ to regulate release [32,70–72].

The endoplasmic reticulum is a major repository for Ca^2+^ inside cells, including the chromaffin cell [28,73,74]. [Ca^2+^]_ER_ is estimated to be in the range of 500–1000 μM. Meanwhile, [Ca^2+^]_cytosol_ is <0.1 μM [28]. This generates an immense chemical gradient favoring Ca^2+^ diffusion into the cytosol from ER. All that is needed is a channel through which Ca^2+^ can flow. Two channels have been invoked as driving Ca^2+^ mobilization downstream of PACAP stimulation. The first is the Ca^2+^-activated, caffeine-sensitive ryanodine receptor (RyR) [32,70,71,75–78]. The other, which we will primarily discuss in this review, is activated by IP3 (and Ca^2+^) — the inositol trisphosphate receptor (IP3R). Three IP3R isoforms (IP3R1, IP3R2, and IP3R3) have been identified in mammalian cells [79]; all three are expressed in chromaffin cells [80]. In the mouse system, the PACAP-stimulated secretory response depends on IP3R1 activity [64,80]. Indeed, when expression of IP3R1 is ‘knocked down’, the Ca^2+^ signals normally evoked by PACAP are reduced [64].

Earlier studies in bovine, rat, and human fetal chromaffin cells identified a role for RyRs in the PACAP pathway [32,70,81]. However, in mouse chromaffin cells, inhibition of RyR function with dantrolene or ryanodine, has little effect on the properties of the Ca^2+^ signals stimulated by PACAP [64]. It is possible that species-specific differences in the organization of the signaling pathways partially accounts for the lack of an effect of RyR inhibition. Mouse chromaffin cells also only express RyR2 and RyR3 mRNAs and at levels that are much lower than mRNA for IP3R1 [64,80].

## Phospholipase Cε is a critical signaling node in the PACAP pathway

**Figure 1.**
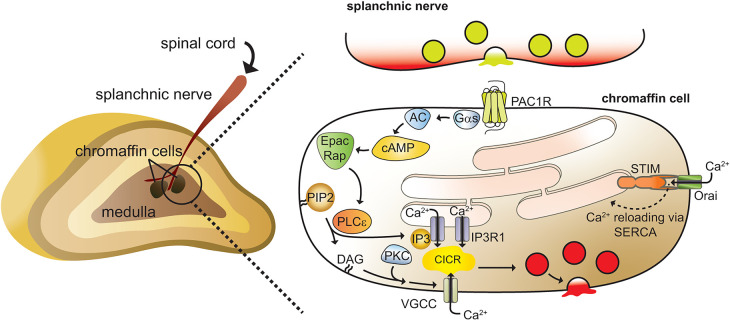
A signaling pathway for PACAP-stimulated exocytosis in chromaffin cells. (Left) The adrenal gland is innervated by sympathetic splanchnic nerves. These nerves form *en passant* synapses with chromaffin cells in the adrenal medulla. (Right) PACAP is released as a result of synaptic vesicle fusion. Subsequent activation of PAC1 receptors stimulates adenylyl cyclase, generates cAMP, and activates PLCε via Epac and Rap. PLCε is a key signaling node in this pathway. PLCε hydrolyzes PIP2 to produce IP3 and DAG. DAG, in turn, activates PKC. PKC may enhance Ca^2+^ entry through L-type Ca^2+^ channels and may also sensitize vesicles to Ca^2+^. Ca^2+^, entering through voltage-gated channels (VGCCs), gates liganded-IP3R1s and enables Ca^2+^ release from the ER (Ca^2+^-induced Ca^2+^ release, CICR). These pathways promote a persistent elevation in intracellular Ca^2+^ that underlies the strong secretory response stimulated by PACAP. ER Ca^2+^ is refilled by Ca^2+^-release activated channels (CRAC) formed by stromal interaction molecule (STIM) and Orai proteins. Once Ca^2+^ enters the cell, it is pumped into the ER by sarcoendoplasmic reticulum Ca^2+^ ATPases (SERCA).

In mouse chromaffin cells, the activation of IP3R1 channels depends on expression and function of phospholipase Cε (PLCε) [82]. As with other members of the PLC family, PLCε hydrolyzes phosphatidylinositol 4,5 bisphosphate (PIP2) to generate IP3 and diacylglycerol (DAG) [82] (Figure 1). PLCε, in turn, is activated downstream of exchange protein activated by cAMP (Epac) and Rap [82]. PACAP is ineffective at evoking either elevations in intracellular Ca^2+^ or exocytosis in PLCε KO cells, although PACAP-stimulated cAMP production is normal [53]. PAC1 receptors have been reported to couple to Gα_s_ and Gα_q_; however, the absolute dependence here on PLCε implicates Gα_s_ in the chromaffin cell signaling pathway [83–85]. According to this model, stimulation of Gα_s_ causes cAMP production and activates Epac [33,53]. Epac then binds to and activates Rap which, in turn, binds directly to PLCε and stimulates the phospholipase [82] (Figure 1). PLCε protein is abundant in the ER [86]. ER-localized PLCε might engage in spatially localized (i.e. compartmentalized) signaling, allowing for efficient and rapid release of Ca^2+^ into the cytosol upon stimulation by Rap [87]. In addition to IP3, PLCε produces DAG which is known to activate PKC [64,88]. Numerous studies have reported the secretory response in chromaffin cells to be regulated by PKC [89–94]. The potential targets of PKC are many: in chromaffin cells, PKC might be involved in altering channel activity (i.e. membrane excitability) and/or the biochemistry of membrane fusion [89–94] (Figure 1).

## ER/PM junctions and their role in PACAP-stimulated exocytosis

As in neurons, the ER network in chromaffin cells extends from the perinuclear region to the PM [64,86,95]. ER-specific probes/proteins such as KDEL and IP3R1 are readily visualized by total internal reflection fluorescence microscopy, which images an ∼100 nm portion of the cell (in *z*) that is delimited by the evanescent field [64]. Moreover, Ca^2+^-rich ‘varicosities’, identified by the bright fluorescence of an ER-targeted GCaMP indicator, are frequently observed abutting the PM [64]. The close spatial relationship that exists between the ER and PM in chromaffin cells is certainly suggestive of a close functional one [96]. For example, Ca^2+^ channels important for triggering action potentials, and, ostensibly, controlling secretion, may be located in close apposition to IP3Rs. Such an organization would be economical from the point of view of the cell. A relatively small Ca^2+^ influx, of the sort known to be stimulated by PACAP, can be greatly amplified due to the action of Ca^2+^ in sensitizing Ca^2+^ release from the ER [79,97,98]. Such an arrangement might underlie the prolonged Ca^2+^ signal evoked by PACAP and the correspondingly long-lived secretory response in the chromaffin cell.

There is precedence for functional coupling of channels on the PM and the ER. For example, in hippocampal neurons, Ca^2+^-induced Ca^2+^ release has been reported at sites where Ca_v_1.3 and RyRs are located in close apposition, presumed to be ER/PM junctions [99]. Together with intermediate conductance Ca^2+^-activated K^+^ channels (KCa3.1), these proteins function to define the properties of the action potential slow afterhyperpolarization. In chromaffin cells, as in neurons, BK and Ca_v_1.3 channels are likely to be co-localized, perhaps even located in clusters [100,101]. Ca^2+^ entry through these voltage-gated channels is presumably important for supplying BK channels with a Ca^2+^ source for reliable Ca^2+^-dependent activation.

We have previously discussed the importance of BK channels and Ca^2+^ channels (especially Ca_v_1.3) for regulating spike properties in chromaffin cells. In neurons of the dorsal cochlear nucleus, Ca^2+^ channels, BK channels, and RyRs were found to be closely localized, possibly to bridge action potential bursts (mediated by Ca_v_s and BK channels) with Ca^2+^-induced Ca^2+^ release from the ER [102]. In the context of PACAP-stimulated secretion, with its multitude of actors, compartmentalization would be expected to improve the efficacy of Ca^2+^ signaling (and exocytosis), as it does in the examples cited above. However, evidence that PAC1 receptors, channels, and/or signaling elements are organized into such compartments in the chromaffin cell has not yet been established.

## Implications for secretion at the synapse

**Figure 2.**
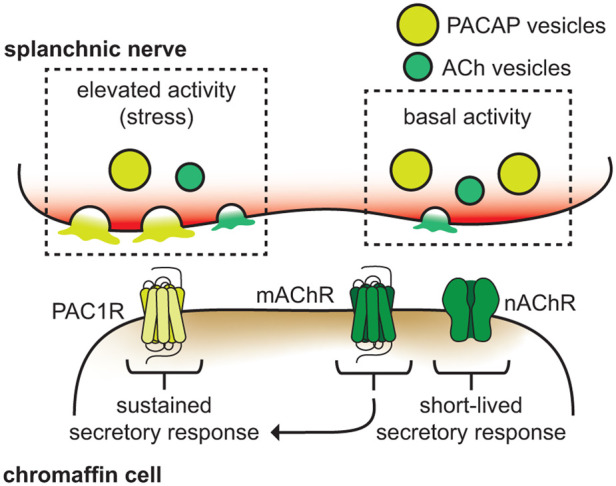
A model for secretion at the splanchnic-chromaffin cell synapse. Different levels of synaptic activity regulate the release of acetylcholine (ACh) and PACAP. ACh, released in response to basal splanchnic activity, binds to and activates nicotinic receptors (nAChRs). This results in a robust, but possibly short-lived secretory response in chromaffin cells, *in situ*. Elevated levels of splanchnic activity preferentially cause release of the neuropeptide transmitter PACAP. PACAP, through PAC1 receptors, stimulates a persistent, non-desensitizing secretory response in chromaffin cells. Muscarinic receptors (mAChRs) may also regulate sustained secretion from chromaffin cells.

It is now firmly established that PACAP, like ACh, is released from splanchnic nerves onto chromaffin cells in the adrenal medulla and causes catecholamine release [27] (Figure 2). However, it is not known if the primary role of PACAP is to augment the secretory stimulus provided by ACh, or if PACAP actually supplants ACh as the principal secretagogue at the splanchnic-chromaffin cell synapse under certain conditions [27]. PACAP does appear to satisfy requirements one might impose on a secretagogue which regulates secretion during stress. Whether it is applied to dispersed chromaffin cells, or released from synaptic endings onto chromaffin cells, *in situ*, PACAP elicits a robust and persistent form of secretion [33,53] (Figure 2). On the other hand, when ACh is continuously applied to cells there is a rundown of the secretory response — a phenomenon attributed to nicotinic receptor desensitization [10,33,103–105]. It should be noted that evidence for nicotinic receptor desensitization, in the setting of elevated splanchnic activity, is mixed [10,106]. Indeed, Parkington and colleagues reported that prolonged periods of repetitive sympathetic nerve stimulation at frequencies up to 30 Hz produced little to no desensitization of nicotinic receptors [106]. Even should nicotinic receptors desensitize, chromaffin cells express multiple subtypes of muscarinic receptors [107,108]. The stimulation of these receptors by ACh or muscarinic agonists is known to evoke a robust secretory response in cells; muscarinic receptors may also contribute to the adrenomedullary secretory response during stress [86,109–112] (Figure 2).

It is possible that the relative contributions of PACAP and ACh to catecholamine secretion, *in vivo*, are more nuanced than we fully appreciate. PACAP may be enriched, for example, in fibers that innervate epinephrine-secreting cells, underlying its role in the counter-regulatory response to hypoglycemia [48]. Alternatively, the release of PACAP versus ACh at adrenal synapses may depend on variations in splanchnic activity. The notion that synaptic release of neuropeptides is activity pattern-dependent certainly has support in the literature [113,114]. What we wish to emphasize here is that although much of this review has focused on the cellular mechanisms underlying PACAP-evoked secretion, the physiological impact of this regulation also remains poorly understood. Indeed, it is hoped that with the emergence of new technologies to measure synaptic function and secretion in the living animal, allied to information gleaned from decades of careful *in vitro* analysis, we will be able to readdress the classical questions that originally motivated research into the function of the adrenal medulla [2,115,116]. As we do so, it will be with a more complete understanding of how cellular exocytosis, of the sort stimulated by PACAP, regulates stress transduction in the periphery.

## Perspectives

- Sympathetic splanchnic nerves deliver the neurotransmitters PACAP and ACh onto adrenal chromaffin cells. In response, chromaffin cells release catecholamine (epinephrine and norepinephrine) and peptide hormones into the bloodstream for circulation throughout the body. These hormones prepare the body for the ‘fight-or-flight’ reaction.
- PACAP binds to and activates PAC1 receptors on chromaffin cells. This triggers a Gα_s_ protein-coupled signaling cascade that results in long-lived elevations in intracellular Ca^2+^ and a strong secretory response from chromaffin cells. The intracellular Ca^2+^ elevations result from a combination of Ca^2+^ influx through voltage-gated, L-type channels on the PM and Ca^2+^ mobilization from the ER. A critical node in this signaling pathway is PLCε.
- Fundamental questions remain regarding the cellular mechanisms by which PACAP evokes secretion. For example, it is not well understood how PAC1 receptors, ion channels, and other proteins are organized to enable efficient signal transduction, increase intracellular Ca^2+^, and trigger exocytosis. Moreover, important signaling intermediates in the PACAP-stimulated secretion pathway likely remain unidentified. PACAP is also not the only neurotransmitter at the splanchnic-chromaffin cell synapse. Thus, it is important to learn whether the synaptic rules governing its release are different from those of ACh, and if PACAP-ergic and cholinergic transmission regulate distinct physiological processes, *in vivo*.

## Abbreviations

ACh: acetylcholine
DAG: diacylglycerol
PACAP: pituitary adenylate cyclase activating polypeptide
PKC: protein kinase C
PLCε: phospholipase Cε
PM: plasma membrane
